# Do Family Caregivers of Dementia Patients Feel Competent in Pain Management?

**DOI:** 10.1093/geroni/igab046.3383

**Published:** 2021-12-17

**Authors:** Hui Zhao, Pamela Kulbok, Ishan Williams, Carol Manning, Rafael Romo

**Affiliations:** 1 James Madison University, Harrisonburg, Virginia, United States; 2 University of Virginia, Charlottesville, Virginia, United States; 3 Dominican University of California, San Rafael, California, United States

## Abstract

Older adults with dementia rely on others to recognize and treat their pain and will ultimately become dependent. Family caregivers (FCGs) play a crucial role in pain management, yet limited data is available regarding the factors that impact their abilities. This qualitative descriptive study sought a deep understanding of FCGs perception of their abilities to manage pain for a loved-one with dementia. A sample of 25 adult family caregivers of community-based older adults with dementia was recruited in central Virginia. Participants were 29 to 95 years old, predominantly white, married, female, and high school graduates. We conducted semi-structured interviews that were audio recorded and analyzed using constant comparative analysis. Participants’ who perceived greater competence with pain management reported less pain for their loved-one, and their level of confidence was influenced by 3 factors: progress and stage of dementia: this increases the complexity of care, affecting FCGs ability to manage pain and engendering a self-perception of incompetence; developing adaptive mechanisms: built self-efficacy and improved FCGs perceived competence;, and support from professionals: a greater degree of support alleviated FCGs concerns and instill new skills, Effective pain management depended on family caregivers’ belief in their own abilities, and perceived competence could be improved by learning new skills or making adaptations. Professional care givers need to routinely assess FCGs abilities and provide adequate interventions.

